# Room-temperature synthesis of near-ultraviolet light-excited Tb^3+^-doped NaBiF_4_ green-emitting nanoparticles for solid-state lighting

**DOI:** 10.1039/c8ra05284k

**Published:** 2018-07-25

**Authors:** Peng Du, Yongbin Hua, Jae Su Yu

**Affiliations:** Department of Electronic Engineering, Kyung Hee University Yongin-si Gyeonggi-do 17104 Republic of Korea jsyu@khu.ac.kr +82 31 201 3820 +82 31 206 2820

## Abstract

We reported a facile reaction technique to prepare Tb^3+^-doped NaBiF_4_ green-emitting nanoparticles at room temperature. Under 378 nm excitation, the prepared samples exhibited the featured emissions of Tb^3+^ ions and the green emission located at 543 nm corresponding to the ^5^D_0_ → ^7^F_4_ transition was observed in the photoluminescence (PL) emission spectra. The PL emission intensity relied on the dopant concentration and its optimum value was determined to be 50 mol%. The involved concentration quenching mechanism was dominated by the electric dipole–dipole interaction and the critical distance was evaluated to be around 10.4 Å. Meanwhile, the color coordinate and color purity of the obtained emission were revealed to be (0.328, 0.580) and 62.4%, respectively. The thermal quenching performance of the synthesized nanoparticles was analyzed using the temperature-dependent PL emission spectra and the activation energy was calculated to be 0.39 eV. By integrating a near-ultraviolet chip with the prepared nanoparticles, a dazzling green light-emitting diode was fabricated to explore the feasibility of the Tb^3+^-doped NaBiF_4_ nanoparticles for solid-state lighting applications.

## Abstract 2

We reported a facile reaction technique to prepare Tb^3+^-doped NaBiF_4_ green-emitting nanoparticles at room temperature.

## Graphical abstract

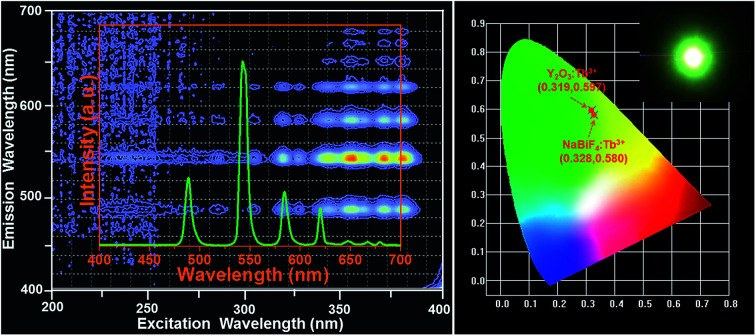

## 1. Introduction

With the increased awareness of environmental protection and energy saving, the phosphor-converted white light-emitting diodes (WLEDs), which are considered as the next generation solid-state lighting and a promising candidate to replace incandescent and fluorescent lamps, have received considerable interest since they can save 50% electricity for illumination.^1–4^ Unfortunately, the commercial strategy of generating white light by utilizing a blue chip to pump the Y_3_Al_5_O_12_:Ce^3+^ yellow-emitting phosphors shows a poor color rendering index and high correlated color temperature due to the lack of a red emission component in the luminescence spectrum.^5,6^ To modify the performance of the WLED device, an alternative technique using a near-ultraviolet (NUV) chip to excite the hybrid (blue-green-red) phosphors was proposed.^7,8^ Herein, developing a highly-efficient single-color phosphor for NUV light excitation is urgent to improve the optical performance of the WLED device.

Currently, the trivalent rare-earth ions doped luminescent materials have been widely developed and employed in various fields including plasma display planes, solid-state lighting, solar cells, biomedicine and temperature detection.^9–13^ Among these trivalent rare-earth ions, the Tb^3+^ ion is being studied extensively as a green-emitting activator because of its dominant green emission arising from the ^5^D_4_ → ^7^F_5_ transition.^14,15^ As is known, the photoluminescence (PL) behaviors of the trivalent rare-earth ions are distinctly affected by the luminescent host material, especially, low phonon energy material since it can improve the possibility of the radiative transition.^16,17^ To date, many inorganics, such as molybdates, silicates, ceramic, fluorides and oxides, were developed as the luminescent host materials for the trivalent rare-earth ions.^18–22^ Compared with other materials, the fluorides, which exhibit a low phonon energy (<350 cm^−1^), are thought of a splendid luminescent host material for trivalent rare-earth ions.^22,23^ Hao *et al.* stated that the NaLnF_4_:Er^3+^/Mg^2+^ nanoparticles did not only exhibit excellent luminescent behaviors but also were potential candidates for tumor diagnosis as a fluorescence probe.^24^ Furthermore, Marciniak *et al.* also revealed that the Nd^3+^-activated NaYF_4_ nanoparticles with strong luminescent performance can be used to detect the temperature.^25^ Note that, to synthesize these rare-earth ions-based fluoride materials, it usually requires lots of expensive rare-earth ions which result in high investment. As a consequence, a new fluoride material should be developed to fix this shortage. Nowadays, the interest in NaBiF_4_ is increasing since the Bi^3+^ ion has some advantages, such as non-toxicity, low cost and intrinsic luminescent properties (*i.e*. absorption band around 300 nm and emission band in the range of 400–500 nm).^26,27^ Zhang *et al.* reported that the NaBiF_4_:Ln^3+^/Yb^3+^ (Ln = Er, Tm, Ho) nanocrystals can emit dazzling upconversion emissions under near-infrared light excitation.^28^ Additionally, we also found that the Eu^3+^-doped NaBiF_4_ nanoparticles were a potential candidate for WLEDs as red-emitting components.^29^ These previous reports confirmed that the NaBiF_4_ is a promising luminescent host material for simultaneous upconversion and down-conversion emissions. Herein, it would be very interesting to investigate the luminescent properties of the trivalent rare-earth ions doped NaBiF_4_ compounds as well as their potential applications. Nevertheless, to the best of our knowledge, the synthesis and photoluminescence (PL) properties of Tb^3+^-doped NaBiF_4_ nanoparticles have not been reported yet. In this work, a series of Tb^3+^-doped NaBiF_4_ nanoparticles were prepared by a simple reaction technique at room temperature, and their phase structure, microstructure and thermal stability as well as PL behaviors were investigated. Ultimately, a green-emitting LED device was package to explore the feasibility of the obtained nanoparticles for solid-state lighting applications.

## 2. Experimental

### 2.1 Synthesis of Tb^3+^-doped NaBiF_4_ nanoparticles

The NaBi_1−*x*_F_4_:*x*Tb^3+^ (NaBiF_4_:*x*Tb^3+^, where *x* = 0.1, 0.2, 0.3, 0.4, 0.5, 0.6 and 0.7) nanoparticles were prepared by a facile room-temperature chemical precipitation method.^28,29^ The powders including NaNO_3_ (99%), Bi(NO_3_)_3_·5H_2_O (99.99%), Tb(NO_3_)_3_·5H_2_O (99.9%) and NH_4_F (99.99%) were used as the raw materials. Briefly, 2 mmol NaNO_3_, (1−*x*) mmol Bi(NO_3_)_3_·5H_2_O and *x* mmol Tb(NO_3_)_3_·5H_2_O were dissolved into 10 ml ethylene glycol (EG) to form solution I. After that, 14 mmol NH_4_F was added into 20 ml EG to prepare solution II. Then, a mixture was achieved by pouring the solution I to the solution II under strong stirring. After 1 min stirring at room temperature, a transparent colloid was formed. Finally, the nanoparticles were obtained through centrifugation, washed with ethanol and de-ionized water several times and then dried at a temperature of 80 °C in air.

### 2.2 Characterization

The prepared samples were characterized by using a Bruker D8 Advance diffractometer, field-emission scanning electron microscope (FE-SEM; LEO SUPRA 5), Thermo Nicolet-5700 Fourier transform infrared (FT-IR) spectrophotometer and fluorescence spectrometer (Scinco FluroMate FS5). The temperature-dependent PL emission spectra of the resultant powders were detected by the fluorescence spectrometer (Scinco FluroMate FS5) and the temperature surrounding the powders was controlled by using a thermocouple (NOVA ST540). The electroluminescence (EL) spectrum of the fabricated LED device was recorded by using a multi-channel spectroradiometer (OL 770).

## 3. Results and discussion

### 3.1 Phase composition and morphology properties

**Fig. 1:**
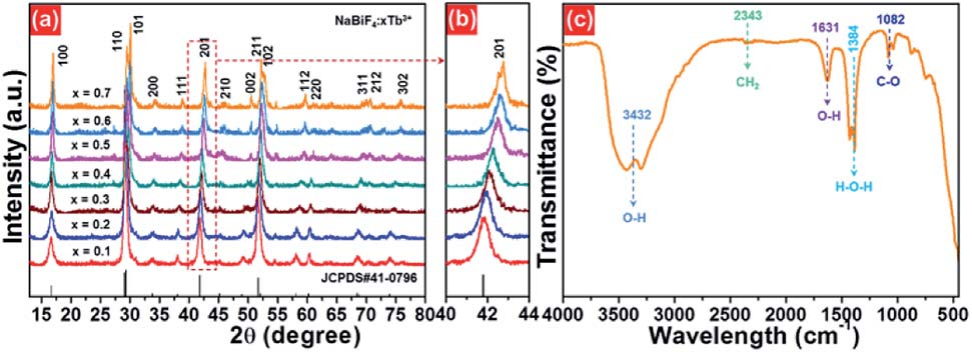
(a) XRD and (b) zoomed XRD patterns of NaBiF_4_:*x*Tb^3+^ nanoparticles as a function of dopant concentration. (c) FT-IR spectrum of the NaBiF_4_:0.5Tb^3+^ nanoparticles.

The X-ray diffraction (XRD) was employed to clarify the phase structure and phase compositions of the studied compounds. The XRD patterns of Tb^3+^-doped NaBiF_4_ nanoparticles prepared at room temperature as a function of dopant concentration are shown in Fig. 1(a) and (b). As presented, the diffraction peaks depicted in Fig. 1(a) matched well with standard NaBiF_4_ (JCPDS#41-0796), implying that all the synthesized compounds consisted of pure hexagonal phase and Tb^3+^ ions were totally entered into the NaBiF_4_ host lattices without changing the host phase structure. Generally, for forming a new solid solution, the radius percentage difference (*D*_r_) between the dopants and potential substituted cations cannot be over 30% and its value can be evaluated by means of the following expression:^3,14^1
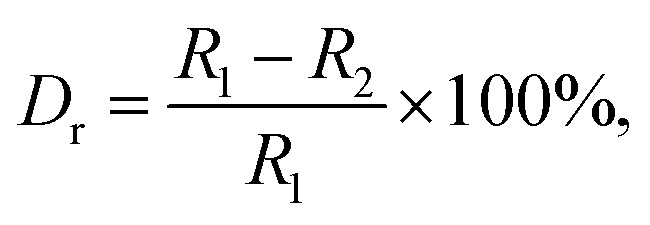
where the parameters including *R*_1_ and *R*_2_ refer to the ionic radii of substituted ions and dopants, respectively. In present work, on account of the designed chemical formula as well as the valences and ionic radii of Bi^3+^ and Tb^3+^ ions, the dopants were anticipated to occupy the places of the Bi^3+^ ions in the NaBiF_4_ host lattices. As a consequence, with the aid of eqn (1) as well as the effective ionic radii of Bi^3+^ (1.03 Å; when coordinate number was 6) and Tb^3+^ (0.923 Å; when coordinate number was 6) ions, the value of *D*_r_ for Bi^3+^/Tb^3+^ couple was revealed to be 10.4% which was much smaller than 30%, implying that the NaBiF_4_:*x*Tb^3+^ compounds can be formed. Furthermore, with elevating the dopant concentration, the zoomed XRD patterns revealed that the diffraction peak gradually moved to the larger angle which was caused by the various ionic radii between the dopants and substituted cations, resulting in the lattice shrinkage (Fig. 1(b)).

To verify the surface functional groups of the final products, the FT-IR spectrum of the representative NaBiF_4_:0.5Tb^3+^ nanoparticles was detected, as shown in Fig. 1(c). In the FT-IR spectrum, the bands at 3432 and 1631 cm^−1^ are related to the O–H stretching vibration, while the sharp peak at 1384 cm^−1^ corresponds to the H–O–H stretching vibration.^30^ Besides, the existence of EG on the surface of the samples is confirmed by the peaks at 2343 cm^−1^ (asymmetric stretching vibration of CH_2_ group) and 1084 cm^−1^ (C–O stretching mode).^31^

**Fig. 2:**
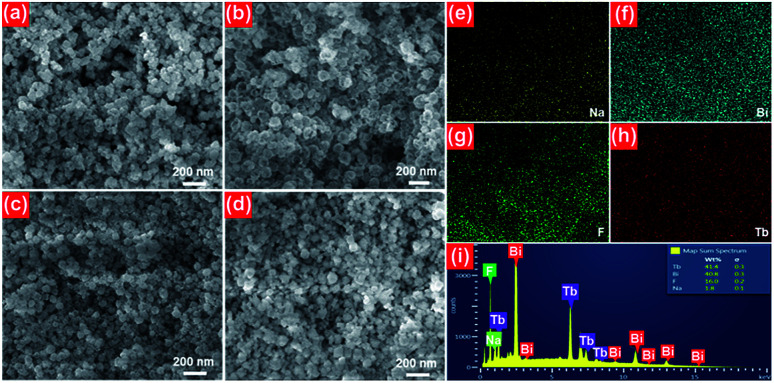
Representative FE-SEM images of the NaBiF_4_:*x*Tb^3+^ nanoparticles: (a) *x* = 0.1, (b) *x* = 0.3, (c) *x* = 0.5 and (d) *x* = 0.7. (e)–(h) Element mapping images and (i) EDX spectrum of the NaBiF_4_:0.5Tb^3+^ nanoparticles sintered at room temperature.

The microstructure behaviors of the final compounds were clarified by the FE-SEM. The FE-SEM images of the NaBiF_4_:*x*Tb^3+^ nanoparticles with various Tb^3+^ ion concentrations are described in Fig. 2. From the FE-SEM images (see Fig. 2(a)–(d)), it is observable that all the synthesized compounds consisted of relatively uniform particles with average size of approximately 100 nm. Moreover, the particle size and shape were hardly varied with increasing the Tb^3+^ ion concentration from 10 to 70 mol%, suggesting that the microstructure of the studied samples is independent of the Tb^3+^ ion concentration. The elemental mapping results revealed that the elements including Na, Bi, F and Tb were evenly distributed over the particles, as presented in Fig. 2(e)–(h). Ultimately, the detection of the Na, Bi, F and Tb peaks in the EDX spectrum, as demonstrated in Fig. 2(i), further verifies that the synthesized samples were made up of Na, Bi, F and Tb.

### 3.2 Room-temperature photoluminescence and energy level diagram

**Fig. 3:**
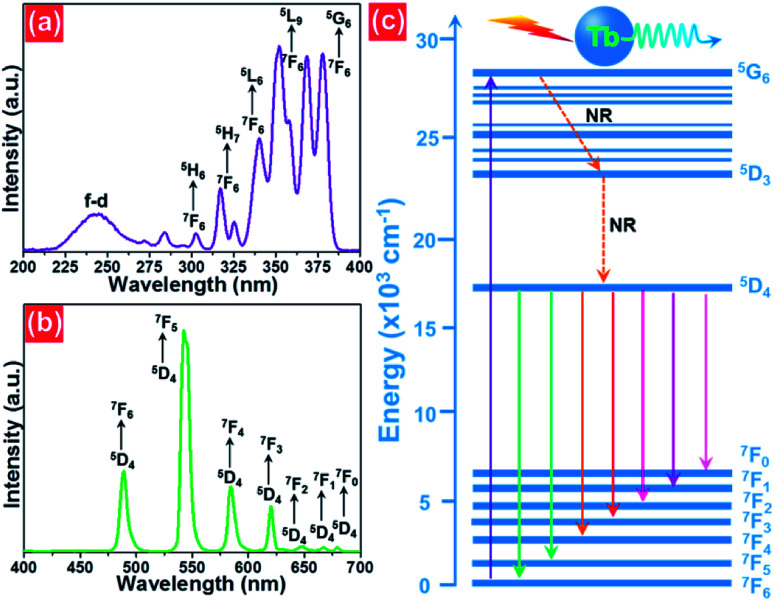
(a) PLE and (b) PL emission spectra of the NaBiF_4_:0.5Tb^3+^ nanoparticles. (c) Simplified energy level diagram of Tb^3+^ ions in the NaBiF_4_:*x*Tb^3+^ compounds.

The room-temperature luminescent performance of the studied samples was characterized by the PL excitation (PLE) and PL emission spectra. The PLE and PL emission spectra of the NaBiF_4_:0.5Tb^3+^ nanoparticles are demonstrated in Fig. 3(a) and (b), respectively. As described in Fig. 3(a), the PLE spectrum monitoring at 543 nm consisted of a weak broad band and several sharp peaks. Particularly, the broad emission band with a central wavelength of at around 250 nm is ascribed to the 4f^8^–5f^7^d^1^ (f–d) transition of Tb^3+^ ions, whereas the other narrow bands at round 302 nm (^7^F_6_ → ^5^H_6_), 317 nm (^7^F_6_ → ^5^H_7_), 340 nm (^7^F_6_ → ^5^L_6_), 352 nm (^7^F_6_ → ^5^L_9_) and 378 nm (^7^F_6_ → ^5^G_6_) are attributed to the intra 4f-4f transitions of Tb^3+^ ions.^32,33^ From the PLE spectrum, one knows that the resultant samples had a strong absorption in the NUV region. This suggests that the NUV light is the proper excitation lighting source for NaBiF_4_:*x*Tb^3+^ nanoparticles. Under the excitation of 378 nm, the typical PL emission spectrum of the NaBiF_4_:0.5Tb^3+^ nanoparticles was recorded and it was composed of several peaks at around 488, 543, 583, 620, 647, 667 and 679 nm corresponding to the intra-4f transitions of Tb^3+^ ions from the ^5^D_1_ ground state to the excited levels of ^7^F_6_, ^7^F_5_, ^7^F_4_, ^7^F_3_, ^7^F_2_, ^7^F_1_ and ^7^F_0_, respectively, as described in Fig. 3(b).^32,33^ For the aim of describing the NUV light-induced visible emission mechanism in NaBiF_4_:*x*Tb^3+^ material system, the simplified energy level diagram of Tb^3+^ ions was molded and shown in Fig. 3(c). As presented, when the Tb^3+^ ions were excited by the NUV light, electrons located at the ground state would be excited to ^5^G_6_ excited level. Then, electrons in the ^5^G_6_ decay to the ^5^D_3_ level and finally to the ^5^D_4_ level by means of the nonradiative (NR) transition processes, as shown in Fig. 3(c). Ultimately, the radiative transitions of ^5^D_4_ → ^7^F_J_ (*J* = 0, 1, 3, 4, 5, 6) occurred and the featured emissions of Tb^3+^ ions were detected in the studied nanoparticles.

**Fig. 4:**
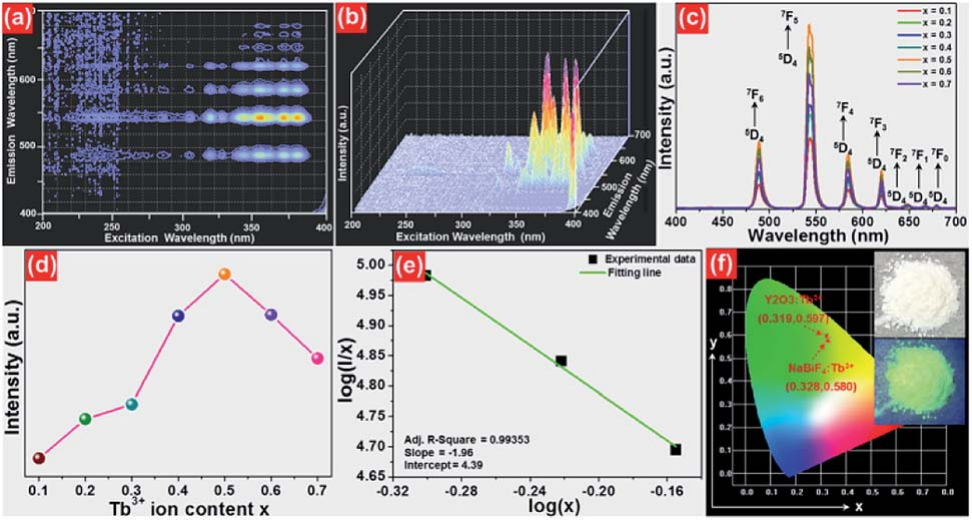
(a) Contour lines and (b) 3D emission spectra of the NaBiF_4_:0.5Tb^3+^ nanoparticles (c) PL emission spectra of the NaBiF_4_:*x*Tb^3+^ nanoparticles. (d) Dependence of PL emission intensity on dopant concentration. (e) Plot of log(*I*/*x*) *vs.* log(*x*). (f) CIE chromaticity diagram of the NaBiF_4_:0.5Tb^3+^ nanoparticles. Inset shows the images of the prepared nanoparticles.

In order to further prove that the resultant nanoparticles can be efficiently pumped by the NUV light, the contour lines and 3D PL emission spectra of the NaBiF_4_:0.5Tb^3+^ nanoparticles, which were detected in the excitation wavelength range of 200–400 nm, were measured and the corresponding results are displayed in Fig. 4(a) and (b), respectively. Obviously, both the contour lines and 3D PL emission spectra exhibited the emissions of Tb^3+^ ions and the intense PL emission intensities were observed in the excitation wavelength range of 350–390 nm, implying that the prepared phosphors can be excited by the NUV light. It is widely accepted that the PL emission intensity of the rare-earth ions is strongly dependent on the dopant concentration. To find out the optimal doping concentration in the studied samples, the Tb^3+^-doped NaBiF_4_ nanoparticles with different dopant concentrations were prepared and their room-temperature luminescent behaviors were characterized by the PL emission spectra. The PL emission spectra of the NaBiF_4_:*x*Tb^3+^ nanoparticles excited at 378 nm as a function of Tb^3+^ ion concentration are depicted in Fig. 4(c). As can be seen in Fig. 4(c), all the compounds showed the characteristic emissions of Tb^3+^ ions and the peak positions were barely affected by the dopant concentration except the emission intensity. It is evident that the PL emission intensity was enhanced with increasing the dopant concentration and maximized at *x* = 0.5. Nevertheless, the concentration quenching, which is caused by the NR energy transfer among the dopants, happened and the PL emission intensity started to decline with further introducing the Tb^3+^ ions into the host lattices, as demonstrated in Fig. 4(d). As is known, to realize the NR energy transfer among the dopants, two different interaction mechanisms of exchange interaction and electric multipole interaction are involved. Besides, the critical distance among the dopants can be used to verify the possible interaction mechanism. In particular, when the critical distance is less than 5 Å, the concentration quenching mechanism among the dopants is dominated by the exchange interaction, whereas the electric multipole interaction contributes to the concentration quenching mechanism when the critical distance is larger than 5 Å.^3,34^ To examine the potential concentration quenching mechanism among the Tb^3+^ ions in the NaBiF_4_ host lattices, the critical distance was calculated using the following equation, given by Blasse:^35^2
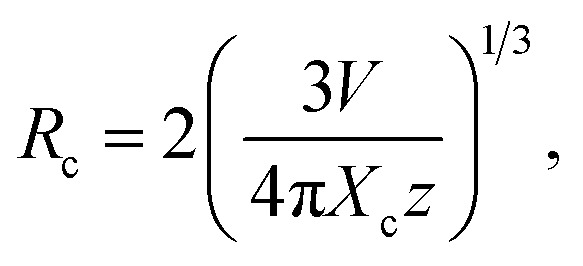
where *R*_c_ is the critical distance, *V* is the volume of the unit cell, *x*_c_ refers to the critical doping concentration and *Z* denotes the number of cations in the unite cell. Here, the values of *V*, *x*_c_ and *Z* for NaBiF_4_ were 140.46, 0.5 and 1.5, respectively. As a consequence, by means of eqn (2), the critical distance of the Tb^3+^ ions in the NaBiF_4_ host lattices was calculated to be approximately 10.4 Å. Since the estimated *R*_c_ value was much larger than 5 Å, it is reasonable to conclude that the electric multipole interaction prevailed in the concentration quenching mechanism.

To further investigate the above concentration quenching mechanism, the relation between the dopant concentration (*x*) and PL emission intensity (*I*) was discussed using the following expression, given by Dexter:^36^3
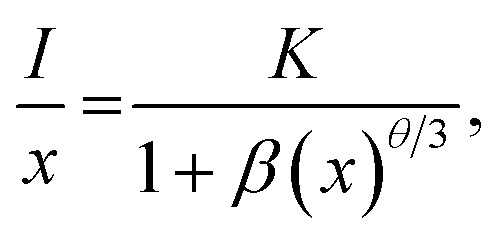
where *k* and *β* are constants and *θ* = 6, 8 and 10 refers to the electric dipole–dipole, dipole-quadrupole and quadrupole–quadrupole interactions, respectively. The plot of log(*I*/*x*) *vs.* log(*x*) for the NaBiF_4_:*x*Tb^3+^ nanoparticles was molded to estimate the *θ* value and the corresponding result is depicted in Fig. 4(e). As presented, the experimental data can be linearly fitted and its slope (*θ*/3) was revealed to be −1.96. Herein, the calculated *θ* value (5.88) was close to 6, implying that the concentration quenching mechanism in the NaBiF_4_:*x*Tb^3+^ nanoparticles was electric dipole–dipole interaction. The Commission Internationale de I'Eclairage (CIE) chromaticity diagram of the NaBiF_4_:0.5Tb^3+^ nanoparticles, which was estimated from the detected PL emission spectrum, is illustrated in Fig. 4(f) so as to evaluate the colorific properties of the resultant samples. It can be seen that the color coordinate of the studied samples was (0.328, 0.580) which was located in the green region. Clearly, this obtained CIE coordinate was very close to the commercial Y_2_O_3_:Tb^3+^ (0.319, 0.597) green-emitting phosphors (see Fig. 4(f)). Furthermore, the prepared nanoparticles can emit visible green light under the irradiation of NUV light, as described in the inset of Fig. 4(f). Apart from the color coordinate, the color purity is another important parameter to describe the colorific behaviors of the obtained phosphors. On the basis of previous literatures,^3,37^ one knows that the color purity can be elevated by the following expression:4
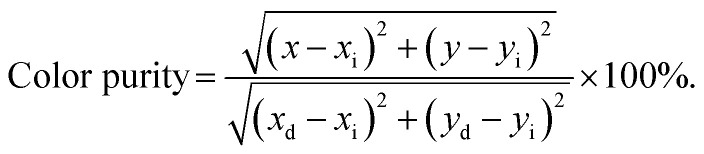
in this expression, the (*x*, *y*), (*x*_i_, *y*_i_) and (*x*_d_, *y*_d_) are related to the CIE coordinates of the NaBiF_4_:0.5Tb^3+^ nanoparticles, white illuminant point and dominant wavelength point, respectively. In present work, (*x*, *y*) = (0.328, 0.580), (*x*_i_, *y*_i_) = (0.310, 0.316) and (*x*_d_, *y*_d_) = (0.251, 0.736) for the dominant wavelength at 543 nm. As a consequence, the color purity of the achieved emission was determined to be about 62.4%.

### 3.3 Thermal stability and electroluminescent property

**Fig. 5:**
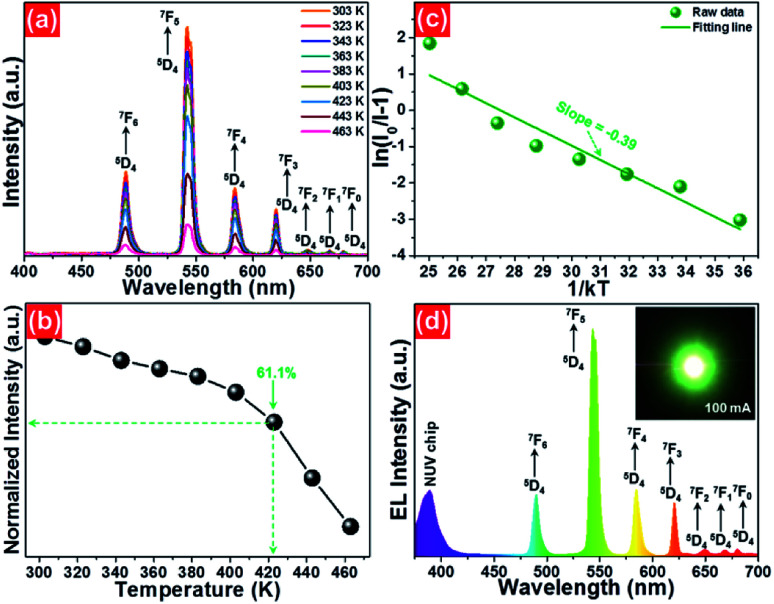
(a) Temperature-dependent PL emission spectra of the NaBiF_4_:0.5Tb^3+^ nanoparticles. (b) Dependence of PL emission intensity on temperature. (c) Plot of ln(*I*_0_/*I* − 1) *vs.* 1/*kT*. (d) EL spectrum of the developed LED device. Inset illustrates the fabricated LED device image.

To evaluate the applicability of phosphors for indoor illumination, their thermal quenching performance should be analyzed since it can directly influence the performance of the packaged LED device. The representative temperature-dependent PL emission spectra of the NaBiF_4_:0.5Tb^3+^ nanoparticles in the temperature range of 303–463 K were detected to examine the thermal stability of the synthesized samples, as illustrated in Fig. 5(a). It is obvious that the emission peaks did not shift with the increment of temperature, whereas the PL emission intensity quenched gradually caused by the thermal quenching effect. Nevertheless, when the surrounding temperature was 423 K, the PL emission intensity of the studied samples still maintained 61.1% of its initial value at 303 K (Fig. 5(b)), indicating that the NaBiF_4_:*x*Tb^3+^ nanoparticles had good thermal stability and were suitable for solid-sate lighting applications. Furthermore, with the aid of following expression, the activation energy (Δ*E*) for the thermal quenching in the NaBiF_4_:*x*Tb^3+^ material system was estimated:^38,39^5
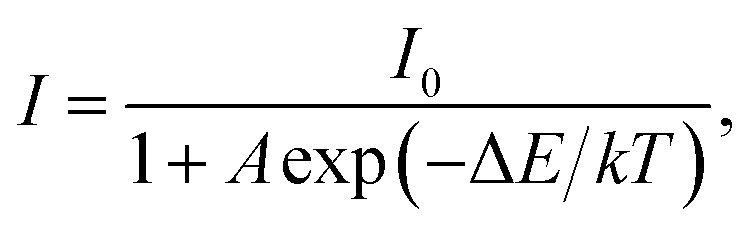
where *I*_0_ and *I* denote the PL emission intensities at initial temperature and *T*, respectively, *A* and *k* refer to constant and Boltzmann coefficient with a fixed value of 8.269 × 10^−5^ eV K^−1^, respectively. From the plot of ln(*I*_0_/*I* − 1) *vs.* 1/*kT* (Fig. 5(c)), it is clear that the experimental data were linearly fitted with a slope of −0.39, demonstrating that the activation energy for the thermal quenching in the NaBiF_4_:*x*Tb^3+^ nanoparticles was 0.39 eV. On the other hand, it was reported that the relation between the activation energy and the possibility of NR transition per unit time (*α*) can be defined as:^40^6
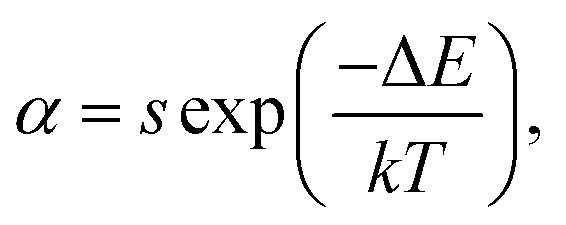
where *s* is associated to the frequency factor. Form the above expression, one knows that higher activation energy results in lower rate of NR transition as well as high thermal stability. In comparison with other synthesized Tb^3+^-doped luminescent materials, such as BiOCl:Tb^3+^ (Δ*E* = 0.31 eV) and Sr_2_Gd_8_Si_6_O_26_:Tb^3+^ (Δ*E* = 0.13 eV),^14,41^ the studied samples exhibited a relatively higher activation energy, further revealing that the Tb^3+^-doped NaBiF_4_ nanoparticles were potential candidates for solid-state lighting.

As a proof of the potential application of the prepared samples for solid-state lighting, a LED device was fabricated using the NaBiF_4_:0.5Tb^3+^ nanoparticles and an NUV chip. In brief, the prepared NaBiF_4_:0.5Tb^3+^ powders were firstly mixed with a silicone epoxy, and then the obtained mixture was coated onto the surface of the NUV chip to package the LED device. After heating at 100 °C for 1 h, the EL emission property of the achieved LED device was examined. Under a basis forward current of 100 mA, the EL spectrum of the developed LED device was measured, as displayed in Fig. 5(d). It can be seen from Fig. 5(d) that the recorded EL spectrum was made up of two parts, that is, a weak band located in the NUV region and series of sharp peaks in the range of 475–700 nm. Especially, the emission band located in the NUV region with a central wavelength of ~385 nm is attributed to the emission of NUV chip and the other sharp peaks 488 nm (^5^D_4_ → ^7^F_6_), 543 nm (^5^D_4_ → ^7^F_5_), 583 nm (^5^D_4_ → ^7^F_4_), 620 nm (^5^D_4_ → ^7^F_3_), 647 nm (^5^D_4_ → ^7^F_2_), 667 nm (^5^D_4_ → ^7^F_1_) and 679 nm (^5^D_4_ → ^7^F_0_) are assigned to the characteristic emissions of Tb^3+^ ions. Ultimately, the fabricated LED device can emit glaring green emission when the injection current was 100 mA (see inset of Fig. 5(d)). These results revealed that the NaBiF_4_:*x*Tb^3+^ nanoparticles can be efficiently excited by the NUV chip and were good green-emitting component candidates for WLEDs.

## 4. Conclusions

In summary, the Tb^3+^-doped NaBiF_4_ green-remitting nanoparticles were synthesized *via* a room-temperature chemical precipitation method. Both the contour lines and 3D PL emission spectra indicated that the NUV light was the proper excitation lighting source for the studied samples. Under 378 nm light irradiation, the synthesized nanoparticles can emit visible green emission. The PL emission intensity was revealed to be dependent on the dopant concentration and the optimal doping concentration for Tb^3+^ ions in the NaBiF_4_ host lattices was 50 mol%. By theoretical calculation, one finds that the electric dipole–dipole interaction can contribute to the concentration quenching mechanism and the critical distance was 10.4 Å. Furthermore, the temperature-dependent PL emission spectra confirmed that the NaBiF_4_:*x*Tb^3+^ nanoparticles also possessed excellent thermal stability. Ultimately, the fabricated LED device, which consisted of a NUV chip and the resultant nanoparticles, revealed that the NaBiF_4_:*x*Tb^3+^ nanoparticles were suitable for solid-state lighting applications as the green-emitting component.

## Conflicts of interest

There are no conflicts to declare.

## Supplementary Material
